# Data-independent acquisition proteomics reveals circulating biomarkers of coronary chronic total occlusion in humans

**DOI:** 10.3389/fcvm.2022.960105

**Published:** 2022-12-05

**Authors:** Jun Li, Xue-Jun Jiang, Qun-Hui Wang, Xing-Liang Wu, Zhe Qu, Tao Song, Wei-Guo Wan, Xiao-Xin Zheng, Xin Yi

**Affiliations:** ^1^Department of Cardiology, Renmin Hospital of Wuhan University, Wuhan, China; ^2^Cardiovascular Research Institute, Wuhan University, Wuhan, China; ^3^Hubei Key Laboratory of Cardiology, Wuhan, China; ^4^Division of Cardiothoracic and Vascular Surgery, Tongji Medical College, Sino-Swiss Heart-Lung Transplantation Institute, Tongji Hospital, Huazhong University of Science and Technology, Wuhan, China; ^5^Key Laboratory of Organ Transplantation, Ministry of Education, NHC Key Laboratory of Organ Transplantation, Chinese Academy of Medical Sciences, Wuhan, China

**Keywords:** data-independent acquisition proteomics, coronary chronic total occlusion (CTO), protein dynamics, percutaneous coronary intervention, human

## Abstract

**Introduction:**

The pathophysiology of coronary chronic total occlusion (CTO) has not been fully elucidated.

**Methods:**

In the present study, we aimed to investigate the potential plasma biomarkers associated with the pathophysiologic progression of CTO and identify protein dynamics in the plasma of CTO vessels immediately after successful revascularization. We quantitatively analyzed the plasma proteome profiles of controls (CON, *n* = 10) and patients with CTO pre- and post- percutaneous coronary intervention (PCI) (CTO, *n* = 10) by data-independent acquisition proteomics. We performed enzyme-linked immunosorbent assay (ELISA) to further confirm the common DEPs in the two-group comparisons (CON vs. CTO and CTO vs. CTO-PCI).

**Results:**

A total of 1936 proteins with 69 differentially expressed proteins (DEPs) were detected in the plasma of patients with CTO through quantitative proteomics analysis. For all these DEPs, gene ontology (GO) analysis and protein-protein interaction (PPI) analysis were performed. The results showed that most of the proteins were related to the negative regulation of proteolysis, regulation of peptidase activity, negative regulation of hydrolase activity, humoral immune response, and lipid location. Furthermore, we identified 1927 proteins with 43 DEPs in the plasma of patients with CTO vessels after immediately successful revascularization compared to pre-PCI. GO analysis revealed that the above DEPs were enriched in the biological processes of extracellular structure organization, protein activation cascade, negative regulation of response to external stimulus, plasminogen activation, and fibrinolysis. More importantly, we generated a Venn diagram to identify the common DEPs in the two-group comparisons. Seven proteins, ADH4, CSF1, galectin, LPL, IGF2, IgH, and LGALS1, were found to be dynamically altered in plasma during the pathophysiological progression of CTO vessels and following successful revascularization, moreover, CSF1 and LGALS1 were validated via ELISA.

**Conclusions:**

The results of this study reveal a dynamic pattern of the molecular response after CTO vessel immediate reperfusion, and identified seven proteins which would be the potential targets for novel therapeutic strategies to prevent coronary CTO.

## Introduction

Chronic coronary total occlusions (CTOs) can be considered the final stage of obstructive coronary artery disease and are associated with soft plaque rupture followed by thrombotic coronary occlusion and organization of thrombotic material (1). The above thrombus is more complexly organized than fresh thrombus formation, with a dense concentration of collagen-rich fibrous tissue at the proximal and distal ends of the lesions, referred to as proximal and distal fibrous caps, respectively, with intervening occluded segments (2). The occluded segment remains biologically active; in particular, the improvement in vascular wall function after CTO revascularization appears to be associated with the restoration of smooth muscle cell function rather than with improved endothelial function or positive remodeling (3). Percutaneous coronary intervention (PCI) for the revascularization of a CTO is a challenging procedure. Unlike acute total occluded vessel immediately after revascularization, ischemia-reperfusion injury after CTO revascularization is associated with myocardial hibernation, a highly vulnerable substrate susceptible to arrhythmias, whereas reperfusion arrhythmias induced after acute vessel revascularization is associated with changes in the intracellular concentration of potassium, sodium, magnesium, and free oxygen radicals (4).

Several proteomics studies have performed comprehensive analyses to recognize the specific patterns and dynamic features of arterial protein networks that constitute the molecular signatures of myocardial ischemia and reperfusion (5, 6). In the ischemic-reperfused myocardium of a pig model with early reperfusion (120 min), proteomic analysis revealed the differential expression of proteins involved in acute-phase response signaling, wound response, nitric oxide production, reactive oxygen species, and glycolysis. These processes reflect an early activation of immunological/inflammatory responses in the post-reperfused myocardium (7). Nakala et al. performed mass spectrometry-based label-free quantification with isolated primary endothelial cells from thrombotic material aspirated from the coronary arteries of patients undergoing treatment for acute ST-segment elevation myocardial infarction (8). The differentially altered protein profiles were related to the metabolism of RNA, platelet activation, signaling and aggregation, cellular responses to stress, and response to elevated platelet cytosolic Ca^2+^ pathways. Elevated production of oxidants, decreased antioxidant biomarkers, and downregulation of proteins with antioxidant properties jointly suggest a role for oxidative stress in mediating endothelial dysfunction during acute myocardial infarction (AMI). Currently, proteomics studies related to myocardial ischemia-reperfusion are mostly performed in AMI patients or animal models, whereas few proteomic studies are involved in the molecular changes occurring in the post-revascularization of coronary CTO.

Collectively, the present study characterizes the dynamic protein changes following successful revascularization in the plasma of coronary CTO patients with a non-acutely occluded vascular segment, which is characteristic of a dense concentration of collagen-rich fibrous tissue or calcification. Moreover, this study attempted to understand the molecular mechanisms of coronary CTO underlying the dynamic process and to provide important data for further studies on coronary CTO.

## Methods

### Patient study and procedures

**Figure 1:**
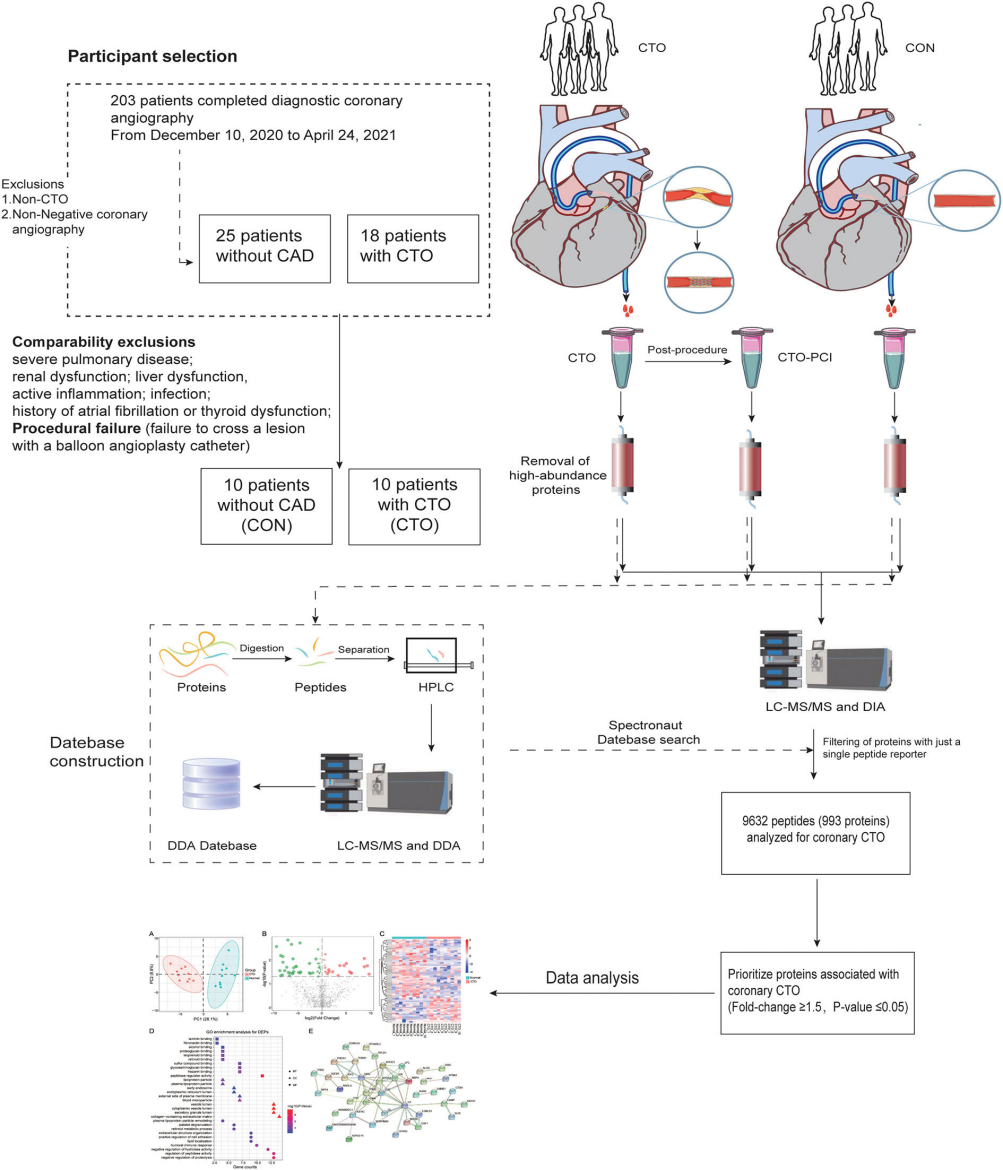
Participant selection and schematic representation of the study design.

The present study was conducted according to the Declaration of Helsinki, and the study protocol was approved by the institutional medical ethics committee. All patients provided informed consent. A total of 203 patients who underwent angiography for symptoms of angina (Canadian Cardiovascular Society (CCS) class 1–3) were recruited consecutively at Renmin Hospital of Wuhan University between November 2020 and July 2021. To avoid the interference of the proteins correlated with other diseases, patients with neurological disease (e.g., cerebral vascular accident), severe pulmonary disease, renal dysfunction, liver dysfunction, active inflammation, infection, coagulation disorders, history of atrial fibrillation, or thyroid dysfunction were excluded. A CTO was defined as complete coronary occlusion of ≥3 months duration with thrombolysis in myocardial infarction (TIMI) grade 0 flow (9). Finally, 10 patients with procedural success were included in the CTO group, defined as angiographic success (final residual stenosis < 30%, with TIMI flow grade ≥2) in the absence of procedural cardiac adverse events (3). After matching for age and sex, 10 patients without significant coronary atherosclerosis served as controls (CON) (Figure 1). In all included CTO vessels, quantitative coronary angiography (QCA) was performed. The reference vessel diameter (RVD) was calculated as the interpolated value of all vessel diameters from the proximal to the distal healthy segments. The occlusion length was measured with bilateral contrast injections. CTO complexity was evaluated using the J-CTO (Multicenter CTO Registry in Japan) score, and the severity of coronary lesions was calculated by the Gensini score (10) (11). In addition, clinical information was collected, including age, sex, height, weight, and histories of smoking, hypertension, and diabetes mellitus. Body mass index (BMI) was calculated as weight (kg) divided by the square of height (m^2^).

### Plasma collection and storage

Coronary blood was withdrawn from the coronary root at the beginning of the cardiac catheterization protocol and the end of revascularization. Blood was transferred into BD Vacutainer SST Plus Blood Collection Tubes, placed on ice, and then centrifuged at 1,500 *g* for 20 min. Plasma was stored at−80°C until use.

### Plasma sample preparation

A 100 μL of sample aliquot was mixed with high abundance removal buffer A at a ratio of 1:3, and the high abundance proteins were removed by Agilent 1,100 HPLC (Agilent, USA) using Multiple Affinity Removal Column Human 14. The depleted plasma samples were concentrated to 250 μL and rapidly transferred to 1.5 mL polypropylene centrifuge tubes. The final protein concentration was measured using a Bradford assay kit (BioRad, Hercules, CA). According to the quantitative results, a 1 μL sample was subsequently taken for SDS-PAGE to verify the high abundance removal. Then, the sample was prepared for protein digestion.

### Protein digestion

The protein samples were digested in a standard sample buffer by the FASP procedure. Briefly, 40 μg of protein in each sample was added to DTT until a final concentration of 100 mM was reached and then heated at 100°C for 5 min. After cooling to room temperature (RT), the mixture was mixed well with 200 μL of UA buffer (pH 8.5) containing 150 mM Tris-HCl and 8 M Urea. All samples were transferred to ultrafiltration with a 30 kDa cutoff membrane filter (Sartorius, Gottingen, Germany) and centrifuged for 30 min at 14,000 × *g* to discard the filtrate. This step was repeated three times. After that, alkylation was performed on the proteins with 100 μL of IAA (50 mM IAA in UA). The protein samples were shaken at 600 rpm for 1 min and incubated under lightproof conditions at 300 rpm for 30 min. Next, each tube was centrifuged at 14,000 × *g* for 30 min, supplemented with 100 μL of ABC buffer (100 mM/L), and centrifuged at 14,000 × *g* for 30 min. Afterward, the sample was added to 100 μL of dissolution buffer (100 mM/L) (Applied Biosystems, USA) and subjected to centrifugation (14,000 × *g*, 30 min) at RT. The above steps were repeated three times. Subsequently, the filtrate was removed, and a thermostat mixer was used for protein digestion with trypsin at 300 rpm and 37°C for 18 h. The tryptic peptides were centrifuged at 14,000 × *g* and RT for 30 min, transferred to a new tube, and then supplemented with 40 μL of 25 mM DS buffer. The tubes were centrifuged at 14,000 × *g* and RT for 30 min for the collection of filtrate. Finally, tryptic peptide quantification at OD280 was conducted.

### HpH

First, 10 μg of each sample's peptides were pooled together. A 1,100 Series HPLC Value System (Agilent) equipped with a Gemini-NX (Phenomenex, 00F-4453-E0) column (4.6 × 150 mm, 3 μm, 110 Å) was used for high-pH reverse-phase HPLC to fractionate peptide samples. Peptides were separated into 25 fractions using a gradient of 4.5–90% ACN in 10 mM ammonium bicarbonate (pH 10) over 51 min. The peptides were then combined into 10 fractions and dried by vacuum centrifugation. Samples were stored at a −80 °C freezer until further analysis.

### Library construction

Ten fractions from Hph HPLC fractionation were reconstituted in 0.1% (v/v) formic acid (FA) in water. Then, 0.2 μL of standard peptides was added to the fractioned sample for subsequent analyses.

### LC-MS/MS analysis-DDA mode

For the construction of the transition library, shotgun proteomics analyses were performed using an EASY-nLCTM 1200 UHPLC system (Thermo Fisher) coupled with an Orbitrap Fusion Lumos mass spectrometer (Thermo Fisher) operating in the data-dependent acquisition (DDA) mode. A sample volume containing 0.5 μg of total peptides from the fractioned sample reconstituted in 0.1% FA was injected into a Thermo Scientific EASY trap column (100 μm × 2 cm, 5 μm, 100 Å, C18). Peptides were separated on a Thermo Scientific analytical column (75 μm × 25 cm, 5 μm, 100 Å, C18) using a 60 min linear gradient from 5 to 100% of eluent B (0.1% FA in 80% ACN) in eluent A (0.1% FA in H2O) at a flow rate of 600 nL/min. The detailed solvent gradient was as follows: 5–28% B, 40 min; 28–90% B, 2 min; and 90% B, 18 min. The Orbitrap Fusion Lumos mass spectrometer was operated in positive polarity mode with a spray voltage of 2.3 kV and capillary temperature of 320°C. Full MS scans ranging from 400 to 1,200 *m*/*z* were acquired at a resolution of 60,000 (at 200 m/z) with an automatic gain control (AGC) target value of 4e5 and a maximum ion injection time of 50 ms. The data-dependent mode was as follows: cycle time, the time between master scan: 3 s. Precursor ions from the full MS scan were selected for fragmentation using higher-energy collisional dissociation (HCD) fragment analysis at a resolution of 15,000 (at 200 m/z) with an AGC target value of 5e4, a maximum ion injection time of 22 ms, a normalized collision energy of 32%, and a dynamic exclusion parameter of 30 s.

### LC-MS/MS analysis-DIA mode

The single sample was reconstituted in 0.1% FA, mixed with 0.2 μL standard peptides (iRT kit, Biognosys), and injected into the EASY-nLCTM 1200 UHPLC system (Thermo Fisher) coupled with an Orbitrap Fusion Lumos mass spectrometer (Thermo Fisher) operating in a data-independent acquisition (DIA) mode. The liquid conditions were the same as above. For DIA acquisition, MS1 resolution was set to 1,20,000 (at 200 *m*/*z*) and MS2 resolution was set to 30,000 (at 200 *m*/*z*). The *m*/*z* range covered from 400 to 1,200 *m*/*z* and variable 40 cycles. The full scan AGC target was set to 4e5 and the IT was set to 50 ms. The DIA settings were an NCE of 32%, a target value of 1e5, and a maximum injection time of 100 ms.

### Data and bioinformatics analysis

Data analysis and visualization of DDA and DIA data were performed using the Proteome Discoverer 2.4 (PD 2.4, Thermo) platform, Biognosys Spectronaut (version 14.9.211124.47784), and persus 1.5. DDA MS raw files were analyzed by PD software (version 2.4) with Mascot 2.3, and peak lists were searched against the protein database. Cysteine carbamidomethylation was set as a fixed modification, and N-terminal acetylation and methionine oxidation were set as variable modifications. The false discovery rate was set to 5% for proteins and peptides and was determined by searching a reverse database. The enzyme specificity was set to trypsin (enabling cleavage before proline), and a maximum of two missed cleavages was allowed in the database search. Peptide identification was performed with an allowed initial precursor mass deviation of up to 10 ppm and an allowed fragment mass deviation of 0.05 Da. MS1-based label-free quantification (LFQ) was performed using the maxLFQ algorithm, and MS2-based label-free quantification was carried out by analyzing DIA raw data using Biognosys Spectronaut (version 14.9.211124.47784) software. Data analysis was carried out as described in Bruder et al. (12) with minor modifications. Briefly, the data extraction and extraction window were set to “dynamic” with a correction factor of 1, and identification was set to a “normal distribution *p*-value estimator” with a *q*-value cutoff of 0.01. The profiling strategy was set to “iRT profiling” with a *q*-value cutoff of 0.01. Ultimately, protein inference was set to “from search engine,” protein quantity was set to “Average precursor quantity,” and the smallest quantitative unit was set to “Precursor ion” (summed fragment ions).

Biological Networks Gene Ontology (BiNGO) 3.03 was used to calculate the gene ontology (GO) term enrichment of differentially expressed proteins (DEPs, defined as quantitative ratio >1.2 or < 0.8 and *p* < 0.05), and protein grouping was analyzed based on functional notes using the GO terms for cellular component (CC), biological process (BP), and molecular function (MF) (13, 14). The Search Tool for the Retrieval of Interacting Genes/Proteins (STRING) software (http://string.embl.de/)

was applied for protein–protein interaction (PPI) analysis. Subsequently, the PPI network was visualized by Cytoscape software (www.cytoscape.org/). Nodes with a higher degree of conne ctivity tend to be more essential in maintaining the stability of the entire network. CytoHubba, a plugin in Cytoscape, was used to calculate the degree of each protein node. In the present study, the top 10 proteins were identified as hub proteins. The Spearman correlation coefficient was used in the correlation analysis, and *t*-tests were carried out to determine the significant differences.

### Enzyme-linked immunosorbent assay

The plasma was defrosted, and the concentrations of proteins were determined using ELISA kits for CSF1 (ELK Biotechnology, Wuhan, China) and LGALS1 (ELK Biotechnology, Wuhan, China). Measurements were performed according to the manufacturer's instructions.

### Statistical analysis

Continuous variables are presented as the mean ± standard deviation. For normally distributed continuous variables, unpaired Student's *t*-test or paired *t*-test were used to assess differences; otherwise, Mann–Whitney U-tests were performed. Categorical variables are presented as frequencies (percentages) and were compared with the chi-square test. For all tests, *p* < 0.05 was considered statistically significant. Statistical analyses were performed with SPSS, version 26 (IBM Corp., Armonk, NY, USA).

## Results

### Patient characteristics

To minimize variability in the small discovery study, patients with CTO (*n* = 10) and CON (*n* = 10) were stringently matched on sex, age, BMI, smoking behavior, the prevalence of diabetes, and hypertension. The mean age of the patients included in this study was 60.1 ± 9.9 years, and 14 individuals (70%) were male (Table 1). There was no significant difference in total cholesterol, low-density lipoprotein (LDL) cholesterol, triglycerides, or apolipoprotein B between these two groups. However, high-density lipoprotein (HDL) cholesterol levels and apolipoprotein A1 (APOa1) levels were significantly lower in the CTO group. As the severity of coronary disease was associated with chronic inflammation, higher white blood cell counts (WBCs), neutrophil counts (NEUs), neutrophil counts, and neutrophil-to-lymphocyte ratios (NEUs) were all observed in the CTO group.

**Table 1:** Baseline clinical characteristics.

	**CON (*n* = 10)**	**CTO (*n* = 10)**	***p*-Value**
Age, y	59.5 ± 10.6	60.7 ± 9.6	0.794
Male, *n*	6	8	0.628
BMI, kg/m^2^	23.85 ± 3.45	23.16 ± 4.84	0.719
Smoke, *n*	4	5	1
Diabetes mellitus, *n*	0	3	0.211
Hypertension, *n*	7	5	0.650
LVEF (%)	58.5 ± 4.7	51.8 ± 11.3	0.068
LVDd, (mm)	43.8 ± 3.0	47.5 ± 4.6	0.075
Triglycerides, mmol/L	1.78 ± 0.97	1.92 ± 2.15	0.247
Total cholesterol, mmol/L	4.77 ± 0.84	3.82 ± 1.21	0.056
HDL cholesterol, mmol/L	1.33 ± 0.39	0.91 ± 0.19	0.005
LDL cholesterol, mmol/L	2.62 ± 0.53	2.28 ± 1.08	0.382
Lipoprotein (a), mg/L	299.1 ± 303.0	180.2 ± 238.6	0.604
sdLDL, mg/L	0.92 ± 0.26	0.82 ± 0.51	0.243
APOa1, g/L	1.26 ± 0.18	1.0 ± 0.13	0.003
APOb, g/L	0.88 ± 0.15	0.77 ± 0.28	0.326
WBC (10^9^/L)	5.13 ± 1.14	7.23 ± 1.90	0.009
NEU (10^9^/L)	3.01 ± 0.64	4.90 ± 1.81	0.009
LY (10^9^/L)	1.65 ± 0.52	1.55 ± 0.52	0.688
NLR	1.94 ± 0.47	3.62 ± 2.37	0.035
PLT (10^9^/L)	208.1 ± 51.0	190.3 ± 32.9	0.366
MONO (10^9^/L)	0.357 ± 0.119	0.589 ± 0.181	0.004
Hb, g/L	139.4 ± 15.8	141.6 ± 18.8	0.780
GFR, mL/min/1.73 m^2^	98.8 ± 18.6	102.2 ± 15.3	0.665
hsCRP, mg/L	0.624 ± 0.643	3.134 ± 3.647	0.182
**Medical treatment**			
Aspirin	3	10	0.003
Clopidogrel/ Ticagrelor	3	10	0.003
Statins	7	10	0.211
ACE/ARB	6	4	0.656
ß-blockers	5	7	0.650
CCB	6	5	1
Nitrates	0	4	0.087

BMI, Body mass index; LVEF, Left ventricular ejection fraction; LVDd, left ventricular end-diastolic dimension; HDL, high-density lipoprotein; LDL, low-density lipoprotein; sdLDL, Small dense low-density lipoprotein-cholesterol; APOa1, Apolipoprotein A1; APOb, Apolipoprotein b; WBC, white blood cell; NEU, neutrophil; LY, lymphocyte; NLR, neutrophil-to-lymphocyte ratio; PLT, platelet; MONO, monocytes; Hb, hemoglobin; eGFR, estimated glomerular filtration rate; hsCRP, High-sensitivity C-reactive Protein; ACE, angiotensin-converting enzyme; ARB, angiotensin receptor blocker; CCB, Calcium Channel Blockers.

The procedural and angiographic characteristics of the CTO group are shown in Table 2. The occlusion length was 31.1 ± 10.1 mm, and the proximal reference vessel diameter and distal reference vessel diameter were 2.62 ± 0.37 and 2.05 ± 0.30 mm, respectively, whereas the stent length was 45.6 ± 19.3 mm. Moreover, the mean J-CTO score was 2.2 ± 0.63 and the Gensini score was 79.1 ± 23.0 in patients in the CTO group. Procedural metrics (contrast volume and total procedural time) are also displayed in Table 2.

**Table 2:** Baseline procedural characteristics.

	**Patient 1**	**Patient 2**	**Patient 3**	**Patient 4**	**Patient 5**	**Patient 6**	**Patient 7**	**Patient 8**	**Patient 9**	**Patient 10**	**Mean ±SD**
Number of diseased vessels	2	3	2	3	3	2	2	1	2	2	2.2 ± 0.63
Target vessel CTO	LCX	LAD	LCX	RCA	RCA	LCX	RCA	LAD	LAD	RCA	
J-CTO score	2	1	2	3	2	2	2	2	3	3	2.2 ± 0.63
Gensini score	72	104	53	74	74.5	101	37	80	112	83	79.1 ± 23.0
Occlusion length (mm)	23.2	27.3	22.3	26.3	28.1	43.9	25.1	28.0	33.0	53.8	31.1 ± 10.1
Proximal RVD (mm)	2.1	3.26	2.47	2.33	2.45	2.41	2.62	2.76	2.70	3.06	2.62 ± 0.37
Distal RVD (mm)	1.85	2.33	1.98	1.72	2.22	1.79	2.28	1.94	1.81	2.64	2.05 ± 0.30
Total stent length (mm)	30	29	24	76.0	54.3	50.2	36	30	49.4	77.3	45.6 ± 19.3
Contrast volume (ml)	202	85	180	167	134	189	96	214	176	153	159.6 ± 43.0
Total procedure time (min)	70	47	58	42	50	64	38	72	64	46	55.1 ± 12.1

CTO, chronic total occlusion; LAD, left anterior descending; LCX, left circumflex; RAD, right coronary artery; RVD, reference vessel diameter.

### Protein profile changes in CTO patients

#### Identifying the differentially expressed proteins

**Figure 2:**
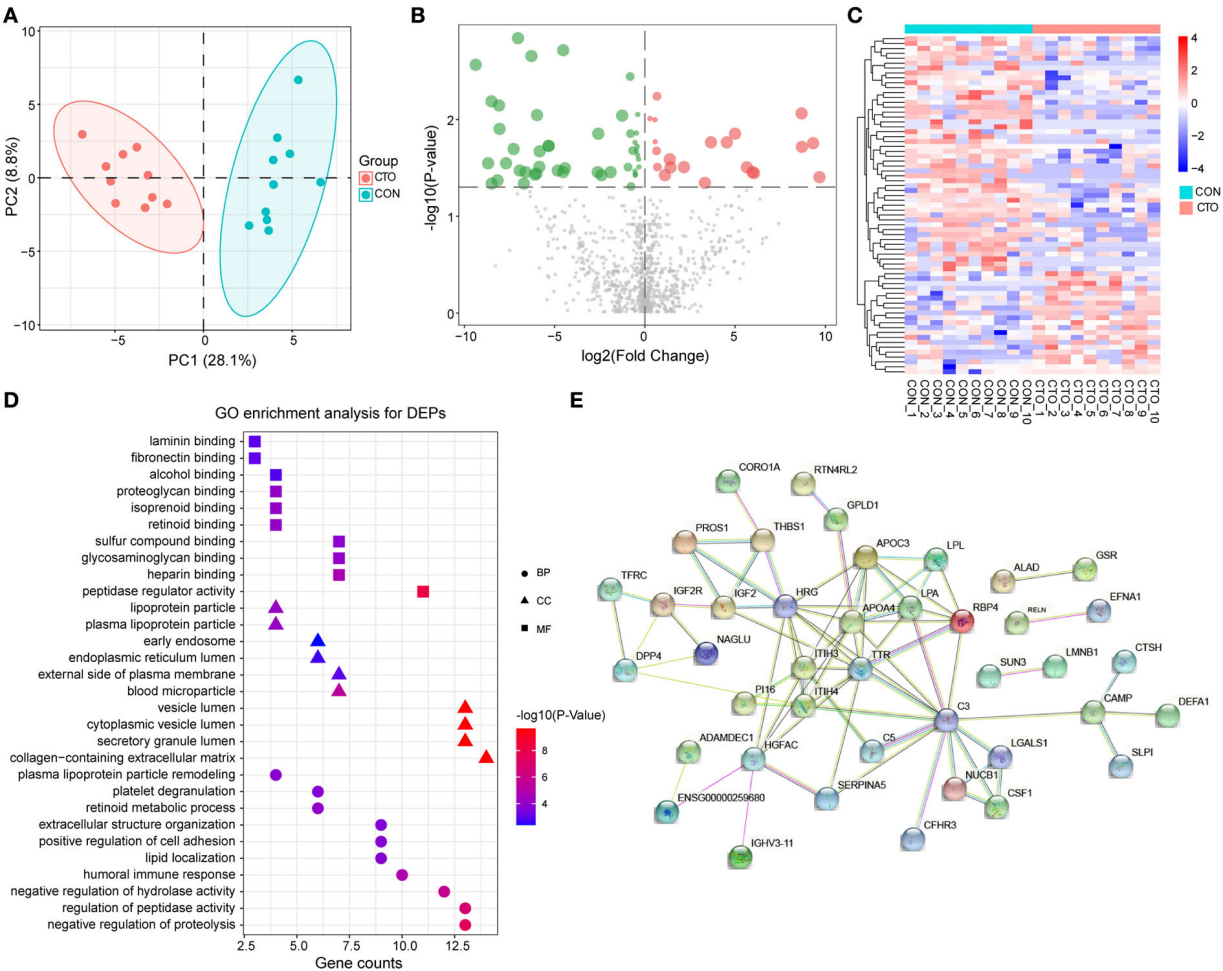
The enrichment analysis shows the DEPs between patients in the CTO and CON groups. **(A)** Principal component analysis distribution of DEPs in CON and CTO plasma samples. **(B)** Volcano plot for DEPs in the CTO and CON groups (green dots represent downregulated proteins, whereas red dots represent upregulated proteins). **(C)** Heatmap of DEPs in the CTO and CON groups. **(D)** Histogram for Gene Ontology (GO) enrichment analysis of DEPs. **(B)** DEPs were classified according to the biological process (BP), molecular function (MF), and cellular component (CC) categories based on Gene Ontology (GO) analysis. **(E)** Protein–protein interaction (PPI) analysis of the DEPs.

To understand the pathophysiologic changes associated with coronary CTO and to identify the major molecular mechanisms involved in the progression of coronary atherosclerosis, it was critical to comprehensively investigate the molecular mechanisms of CTO at the protein level. Therefore, we performed a DIA proteomics study to compare the circulating protein profile differences between CTO and CON patients. It was shown that 973 proteins in the CTO group and 963 proteins in the CON group were identified. Principal component analysis (PCA) further validated the clear distinction between the two groups, suggesting that as coronary atherosclerosis progresses, plasma protein expression patterns were significantly different in the CTO patients and CON patients (Figure 2A). Of the identified proteins, 69 proteins exhibited significant down- or upregulation in the patients with coronary compared to the CON subjects, which indicates that those DEPs might be involved in the biological process of progression of coronary arteries from normal vessels to CTO. Volcano plots presented the proteins in a graph of p-values according to a given statistical test vs. fold change (Figure 2B). A heatmap reflected the protein expression values in different groups and functional cluster analysis of differentially expressed proteins (Figure 2C). Proteins with similar functions have a relatively shorter Euclidean distance. The red and green colors represent up- and downregulated proteins, respectively. The top 20 DEPs in plasma between patients in the CON and CTO groups are summarized in Table 3.

**Table 3:** Top 20 differential expressed proteins in plasma of CTO vs. CON.

**Protein uniprot ID**	**Protein name**	**Gene name**	**Log_2_FC (CTO vs. CON)**	**Adjusted *P-*value (CTO vs. CON)**	**Peptides**	**Function**
D6RAR4	Hepatocyte growth factor activator	HGFAC	9.67003	0.0394	1	Serine-type endopeptidase activity
P00390	Glutathione reductase,	GSR	−9.373	0.0027	3	Electron transfer activity
A8K6C9	Insulin-like growth factor II (Preptin)	IGF2	9.32043	0.0175	2	Growth factor activity
A8K7Q1	Nucleobindin-1	NUCB1	8.69996	0.0191	5	Calcium ion binding
B2MUX6	Insulin-like growth factor 2	IGF2	−8.6843	0.0283	1	Growth factor activity
A0A5C2G5L7	IGL c3870_light_IGKV1-33_IGKJ3 (Fragment)		8.68357	0.0086	1	
A0A5C2G5F8	IGH c160_heavy__IGHV1-69_IGHD3-9_IGHJ6 (Fragment)	ENSG00000278782	−8.4998	0.0064	2	
A0A384ME06	Epididymis secretory sperm binding protein	EFNA1	−8.4862	0.0459	1	Ephrin receptor binding
Q86SQ4	Adhesion G-protein coupled receptor G6	ADGRG6	−8.2273	0.0081	3	Collagen binding
A0A1B1RVA9	Lipoprotein lipase	LPL	−8.1727	0.0091	3	1-acyl-2-lysophosphatidylserine acylhydrolase activity
M0R1Q1	Complement C3 (Fragment)	C3	−8.1644	0.0425	1	
A0A5C2FZ05	IGL c1470_light_IGKV6D-21_IGKJ1 (Fragment)		−8.0822	0.0071	2	
A0A449C188	IGLV2-14*01_S1338 (Fragment)	IGLV2-14	−8.0536	0.0125	1	
A0A0S2Z421	Myocilin (Fragment)	MYOC	−7.6942	0.0283	4	Receptor tyrosine kinase binding
A0A5C2GQ05	IG c1476_heavy_IGHV3-7_IGHD4-11_IGHJ4 (Fragment)	IGHV3-43D	−7.2739	0.0201	2	
A0A384MR27	Galectin	LGALS1	−7.0301	0.0014	1	Identical protein binding
A0A024R693	Galectin	hCG_22119	−6.9338	0.033	4	Carbohydrate binding
A0A024R877	Delta-aminolevulinic acid dehydratase	ALAD	−6.7073	0.0459	1	Metal ion binding
A0A140T971	Dimethylargininase (Fragment)	DDAH2	−6.6125	0.0349	1	Dimethylargininase activity
A0A024R0A1	Macrophage colony-stimulating factor 1	CSF1	−6.2827	0.0022	3	Cytokine activity

#### Functional analysis of differentially expressed proteins

After identifying the DEPs in plasma from patients with CTO compared with that of patients with CON, the associated molecular functions and biological processes were further explored. The 69 DEPs were classified using GO annotation. As shown in Figure 2D, our results revealed that the main biological processes (BP) of these proteins were related to the negative regulation of proteolysis, regulation of peptidase activity, negative regulation of hydrolase activity, humoral immune response, and lipid location. Additionally, the main molecular functions (MF) of these DEPs were focused on peptidase regulator activity, heparin-binding, glycosaminoglycan binding, sulfur compound binding, and retinoid binding. Moreover, the cellular components (CC) of these proteins were mainly located in the collagen-containing extracellular matrix, secretory granule lumen, cytoplasmic vesicle lumen, vesicle lumen, and blood microparticle.

To further systematically analyze the possible regulated signaling network associated with the altered proteomes of patients with coronary CTO, STRING tools were used to generate protein–protein interaction networks for all the differentially expressed proteins. Our results indicated that a total of 60 nodes and 97 edges were involved in the protein–protein interaction network (Figure 2E).

### Protein profile changes in CTO patients after successful revascularization

#### Identifying the differentially expressed proteins

**Figure 3:**
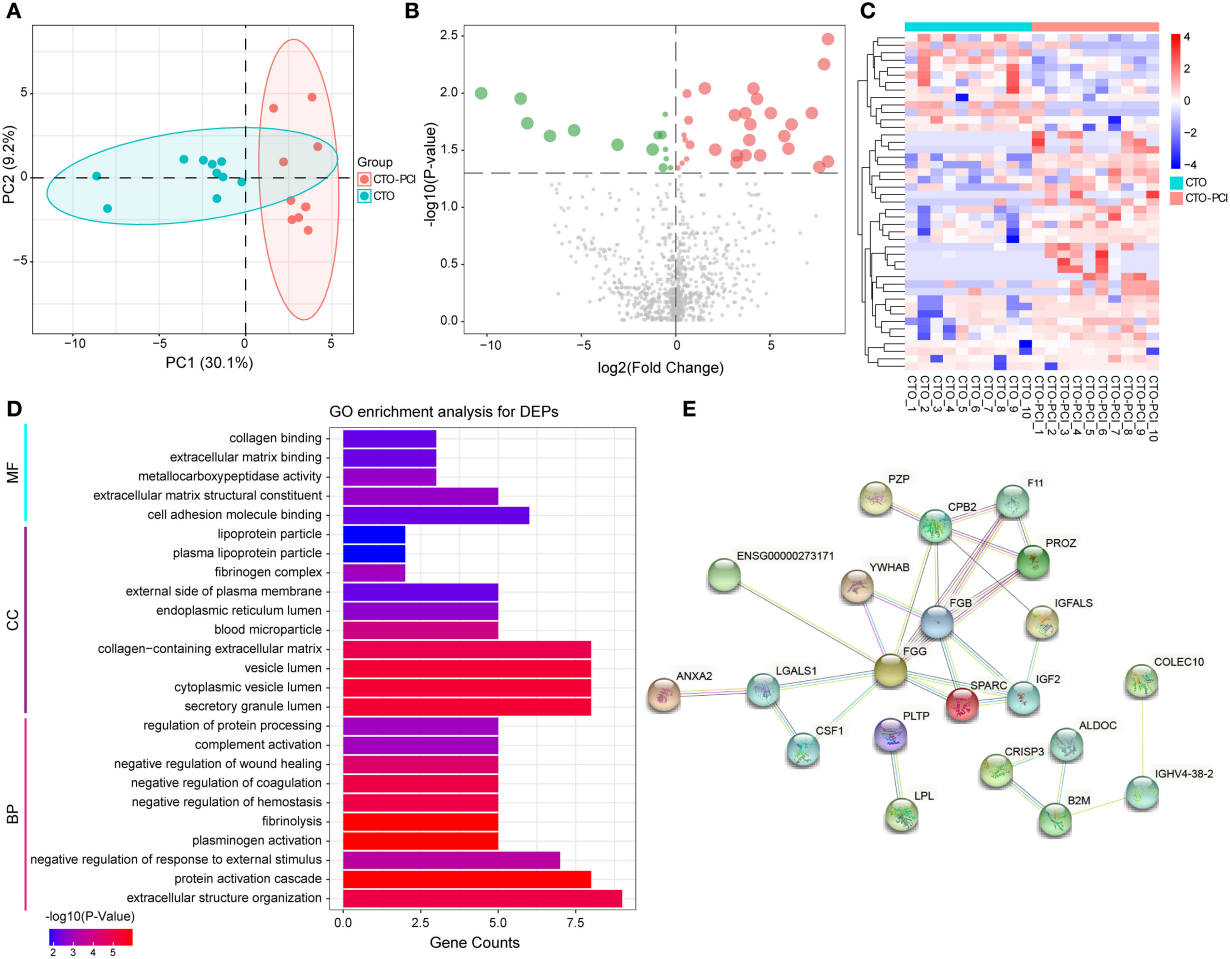
The enrichment analysis shows the DEPs between the CTO and CTO post-PCI (CTO–PCI) groups. **(A)** Principal component analysis distribution of DEPs in patients with CTO and their post-PCI plasma samples. **(B)** Volcano plot for DEPs in patients with CTO and those post-PCI (green dots represent downregulated proteins whereas red dots represent upregulated proteins). **(C)** Heatmap of DEPs in patients with CTO and those post-PCI. **(D)** Histogram for Gene Ontology (GO) enrichment analysis of DEPs. **(B)** DEPs were classified according to the biological process (BP), molecular function (MF), and cellular component (CC) categories based on Gene Ontology (GO) analysis. **(E)** Protein–protein interaction (PPI) analysis of the DEPs.

To further understand the pathological changes associated with CTO before and after revascularization, we took a target CTO coronary blood by guiding the catheter immediately after PCI and then identified the proteomic profile changes related to CTO after successful revascularization. Our results indicated that a total of 954 proteins were identified in this cohort. Of them, 43 proteins exhibited significant down- or upregulation when compared to pre-PCI. PCA showed important segregation between the CTO and CTO–PCI subjects, indicating that immediate restoration of coronary blood flow in an occluded coronary artery could cause significant changes in intracoronary proteomics (Figure 3A). To further illustrate the qualified and dysregulated proteins, volcano plots (Figure 3B) and a heatmap (Figure 3C) were generated. There were 29 upregulated and 14 downregulated proteins that showed a clear separation between the CTO and CTO-PCI individuals. The top 20 differentially expressed proteins in plasma between the CTO and CTO-PCI groups are shown in Table 4.

**Table 4:** Top 20 Differential expressed proteins in plasma of CTO-PCI vs. CTO.

**Protein uniprot ID**	**Protein name**	**Gene name**	**Log_2_FC (CTO-PCI vs. CTO)**	**Adjusted *P*-value (CTO-PCI vs. PCI)**	**Peptides**	**FUNCTION**
A0A1B1RVA9	Lipoprotein lipase	LPL	8.069106	0.0033	3	1-acyl-2-lysophosphatidylserine acylhydrolase activity
A0A5C2GU73	IG c857_light_IGLV3-21_IGLJ2 (Fragment)		8.065894	0.0396	2	
A0A140VK46	Proteasome subunit beta	PSMB4	7.858863	0.0055	2	Lipopolysaccharide binding
A0A5C2GD53	IGH + IGL c19_heavy_IGHV3-21_IGHD1-26_IGHJ3 (Fragment)	IGHV3-11	7.603366	0.0443	1	
A0A024QZ64	Fructose-bisphosphate aldolase	ALDOC	7.209727	0.0149	1	Fructose-bisphosphate aldolase activity
A0A5C2G5F8	IGH c160_heavy__IGHV1-69_IGHD3-9_IGHJ6 (Fragment)	ENSG00000278782	7.163619	0.0289	2	
A0A5C2GA94	IGH + IGL c122_light_IGKV4-1_IGKJ3 (Fragment)		6.134675	0.0187	2	
O43157	Plexin-B1	PLXNB1	5.97339	0.0305	2	GTPase activating protein binding
A0A5C2FZ05	IGL c1470_light_IGKV6D-21_IGKJ1 (Fragment)		5.752151	0.0237	2	
M0QZL2	Multiple epidermal growth factor-like domains protein 8 (Fragment)	MEGF8	5.035202	0.0149	1	
A0A5C2GLE5	IG c1000_light_IGKV3-20_IGKJ2 (Fragment)		4.932223	0.0311	2	
B5BU24	14-3-3 protein beta/alpha	YWHAB	4.472087	0.0349	1	
B2R6W4	Frizzled-related protein 1	FRZB	4.306862	0.0112	3	Extracellular region
Q8IUX7	Adipocyte enhancer-binding protein 1	AEBP1	4.116601	0.0090	5	Calmodulin binding
A0A0B6XK00	Lectin, galactoside-binding, soluble, 1 (Fragment)	LGALS1	3.953884	0.0187	2	
A0A286YFJ8	Immunoglobulin heavy constant gamma 4 (Fragment)	IGHG4	3.900372	0.0256	3	Integral component of membrane
A0A024RDF8	Alcohol dehydrogenase 4	ADH4	3.699966	0.0349	2	Alcohol dehydrogenase activity, zinc-dependent
Q6NUJ1	Proactivator polypeptide-like 1	PSAPL1	3.696426	0.0149	1	Cytosol
A0A024R0A1	Macrophage colony-stimulating factor 1	CSF1	3.24073	0.0349	2	Cytokine activity
A0A384MR27	Galectin	LGALS1	3.222138	0.0403	1	Identical protein binding

#### Functional analysis of differentially expressed proteins

After identifying the differentially expressed proteins in plasma from patients with CTO compared with that from CTO after PCI, the associated molecular functions and biological processes were further discussed. The 43 DEPs were classified using GO annotation (Figure 3D). BP analysis showed that the DEPs were dramatically enriched in extracellular structure organization, protein activation cascade, negative regulation of response to external stimulus, plasminogen activation, and fibrinolysis. MF analysis showed that the DEPs were significantly related to cell adhesion molecule binding, extracellular matrix structural constituent, metallocarboxypeptidase activity, extracellular matrix binding, and collagen binding. CC analysis showed that the DEPs were significantly enriched in the secretory granule lumen, cytoplasmic vesicle lumen, vesicle lumen, collagen-containing extracellular matrix, and blood microparticle. To further systematically explore the possible regulated signaling network associated with the altered proteomes of coronary CTO patients after PCI, our results indicated that a total of 39 nodes and 31 edges were involved in the protein–protein interaction network by STRING tools (Figure 3E).

### Identification and validation of the common DEPs for the two comparisons (CON vs. CTO and CTO vs. CTO–PCI)

**Figure 4:**
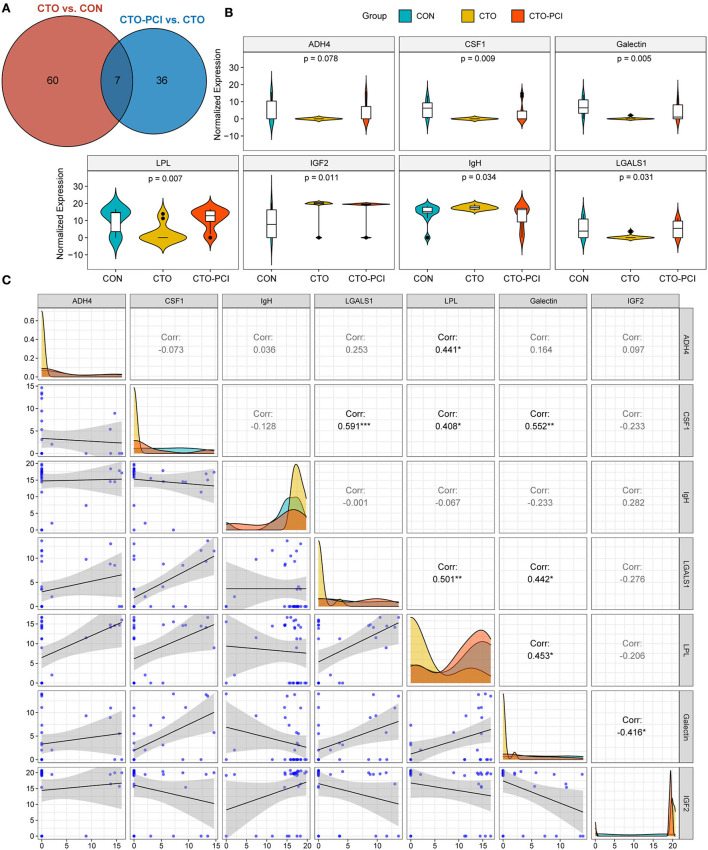
The common DEPs for the two comparisons (CTO vs. CON and CTO vs. CTO-PCI). **(A)** Venn diagram showing the distribution of common DEPs in CTO vs. CON and CTO vs. CTO-PCI (*p* < 0.05). **(B)** Violin plot showing the distribution of common DEPs in each group. **(C)** Correlation analysis showed the internal connection of protein levels among the common DEPs (*p < 0.05, **p < 0.05, ***p < 0.001).

To investigate the potential proteins associated with the biological processes of CTO pathological progression and coronary CTO revascularization, we generated a Venn diagram to identify the common DEPs in the two-group comparisons (CON vs. CTO and CTO vs. CTO-PCI) (Figure 4A). Finally, it was found that seven proteins, ADH4, CSF1, galectin, LPL, IGF2, IgH, and LGALS1, were dramatically regulated and significantly different between the two groups (Table 5). To disclose the underlying mechanism by which these DEPs influenced the prognosis of CTO, we further explored the correlations between the seven DEPs and the severity of CTO lesions with the fruitful achievements in PCI in the field of coronary CTO lesions. Violin plot analysis showed that the levels of ADH4, CSF1, galectin, LPL, and LGALS1 in the CTO group were lower than those in the CON and CTO-PCI groups, whereas the levels of IGF2 and IgH in the CTO group were higher than those in the other two groups (*P* < 0.05) (Figure 4B). Then, we conducted a correlation analysis among these seven DEPs. Intriguingly, ADH4 was positively correlated with LPL (*R*^2^ = 0.441, *p* < 0.01). CSF1 had significant positive correlations with LGALS1 (*R*^2^ = 0.591, *p* < 0.001), LPL (*R*^2^ = 0.408, *p* < 0.05) and galectin (*R*^2^ = 0.552, *p* < 0.01). LGALS1 was positively correlated with LPL (*R*^2^ = 0.501, *p* < 0.01) and galectin (*R*^2^ = 0.442, *p* < 0.05). LPL was positively correlated with galectin (*R*^2^ = 0.453, *p* < 0.01). The opposite trend was observed between galectin and IGF2 (*R*^2^ = −0.416, *p* < 0.05) (Figure 4C).

**Table 5:** The common DEPs for the two comparisons.

**Protein uniprot ID**	**Protein name**	**Gene name**	**Peptides**	**Function**
A0A024RDF8	Alcohol dehydrogenase 4	ADH4	2	Alcohol dehydrogenase activity
A0A024R0A1	Macrophage colony-stimulating factor 1	CSF1	2	Cytokine activity
A0A2U8J8R0	Ig heavy chain variable region (Fragment)	IgH	4	
A0A0B6XK00	Lectin, galactoside-binding, soluble, 1 (Fragment)	LGALS1	2	An evolutionarily conserved β-galactoside-binding lectin
A0A1B1RVA9	Lipoprotein lipase (LPL) (EC 3.1.1.34)	LPL	3	1-acyl-2-lysophosphatidylserine acylhydrolase activity
A0A384MR27	Galectin	LGALS1	1	Identical protein binding
A8K6C9	Insulin-like growth factor II (Preptin)	IGF2	2	Growth factor activity

**Figure 5:**
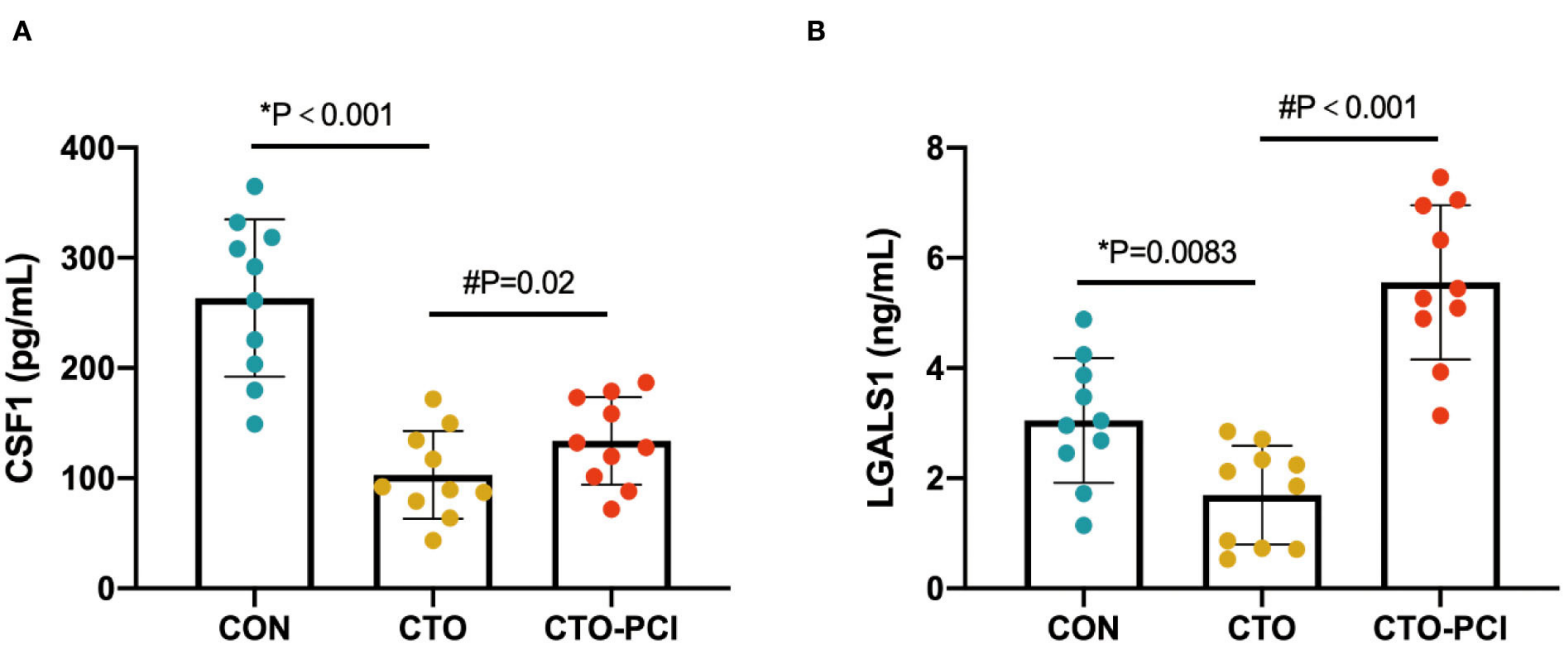
ELISA-based validation of two randomly selected proteins of the common DEPs. **(A, B)** ELISA analysis of CSF1 and LGALS1 in the plasma of different groups (*unpaired t-test; #paired *t*-test).

To further increase the reliability of the results, two proteins (CSF1 and LGALS1) were randomly selected for validation using ELISA. The results showed that the expression pattern of the two proteins was consistent with that in the initial proteomics study (Figure 5). Collectively, these findings suggested that this seven-protein signature may play an important role in the progression of coronary CTO and the future prevention of coronary diseases.

## Discussion

In a previous study, proteomic analysis of plasma from patients with triple vessel CAD who underwent coronary artery bypass grafting (CABG) surgery was conducted (15). A total of 76 DEPs were identified in the plasma of CAD patients and were involved in different physiological processes, including coagulation, platelet activation, the complement pathway, and the Wnt/Fz signal-transduction pathway. This result indicated that the above-mentioned DEPs might play an essential role in the progression of CAD. The present study was the first to investigate the changes in the proteome profile of intracoronary plasma in 10 patients with CTO vessels and 10 CONs matched by sex, age, BMI, smoking behavior, the prevalence of diabetes, and hypertension. Dyslipidemia is the most important risk factor for atherosclerosis. The lipid levels (total cholesterol, LDL cholesterol, triglycerides, and apolipoprotein b) were similar between the two groups. Perhaps the CTO groups had started lipid-lowering therapy when they first experienced angina symptoms. HDL and APOa1 (the major protein constituent of HDL cholesterol) levels were significantly lower in the CTO group, which might be related to the weak effect of statins on HDL cholesterol (generally < 10% increase) (16, 17). Chronic inflammation is a key feature of atherosclerosis. Epidemiologic studies have shown that the WBC count and neutrophil-to-lymphocyte ratio (NLR) are independent risk factors for future cardiovascular events in patients with atherosclerosis (18, 19). The blood cell features of the CTO patients included in our study were also consistent with the above studies. Finally, our results showed that 69 proteins were identified and had significantly different expression levels between the CTO patients and the CON subjects. The top five significant biological process terms were enriched in the negative regulation of proteolysis, regulation of peptidase activity, negative regulation of hydrolase activity, humoral immune response, and lipid location. The DEPs identified in this study were not enriched in the chronic inflammatory pathway, which may be related to the fact that the proteins were derived directly from intracoronary arterial blood rather than venous blood.

This was the first comprehensive proteomics analysis of molecular changes in the plasma of patients with coronary CTO at early post-PCI. The mean procedure time for CTO patients in the present study was 55.1 ± 12.1 min. After early coronary reperfusion, a total of 43 DEPs were identified in coronary CTO by DIA quantification compared to pre-PCI. GO analysis indicated that the DEPs were associated with biological processes, including extracellular structure organization, protein activation cascade, negative regulation of response to external stimulus, and plasminogen activation. Previous studies demonstrated that the changes in oxidized phospholipids, lipoprotein(a), and biomarkers of oxidized lipoproteins in plasma were different between CTO coronary arteries and non-CTO after immediately successful PCI (20, 21). In addition, a sharp increase in the levels of oxidized phospholipids/APOB and LPA was observed in non-CTO vessels following PCI. However, the above changes were in CTO vessels following PCI. These results indicated that oxidized phospholipids released from the non-CTO vessel wall might contribute to the disruption and embolization of plaque content. However, PCI of CTO vessels might not induce the above release. It is well-known that the local vascular imbalance between oxidative stress and antioxidative stress plays critical roles in vascular damage and the increased proinflammatory status and then promotes an imbalance of the local vascular pro- and anti-inflammatory response and leads to coronary atherogenesis (22, 23). However, similar to a previous study, we did not identify any DEPs associated with oxidative stress and the inflammatory response in CTO vessels after successful PCI (21). Interestingly, the altered proteins in coronary CTO after successful PCI in our study were enriched in the biological process of extracellular structure organization by GO analysis. We hypothesize that the reason for this is that the thrombus in the chronically occluded vessel segment has a more complex composition, and that long-term thrombus organization leads to increased vascular extracellular matrix content, those DEPs circulating levels increased when the renewal of antegrade flow into a previously occluded artery (24, 25).

Another important finding of the present study was the identification of seven proteins (ADH4, CSF1, galectin, LPL, IGF2, IgH, and LGALS1) that might be dynamically altered in plasma during the pathophysiological progression of CTO vessels and following successful revascularization. LPL is a principal enzyme in lipoprotein metabolism, tissue lipid utilization, and energy metabolism (26). In the heart, the majority of LPL is synthesized in cardiomyocytes and secreted onto the cell surface. In response to glucose and endothelial cell heparinase secretion, LPL can move to the vascular lumen (27). More studies have shown that pre-heparin LPL mass negatively correlates with insulin resistance, which has been suggested as a biomarker for metabolic syndrome and related cardiovascular diseases (28, 29). Similarly, our results suggested that increased LPL in the plasma of CTO was downregulated after PCI, which might be a useful predictor of the severity of coronary CTO lesions. After revascularization of coronary CTO and reperfusion of cardiomyocytes, most of the LPL might be released into circulation under the status of the coronary stent against the luminal side of the endothelium. Macrophage CSF1 is a cytokine and a hematopoietic growth factor that regulates macrophage survival, differentiation, proliferation, and migration from precursor hematopoietic stem cells (30). Multiple studies have shown that CSF1 can be actively expressed in atherosclerotic lesions and plays a role in atherosclerosis formation (31, 32). However, in the present study, it was found that plasma CSF1 level was the lowest in CTO patients and was high in the CON group or after CTO vessel revascularization. This was perhaps related to the isolation of atherosclerotic plaques of CTO lesions from circulating blood. LGALS1, also named galectin-1, is an evolutionarily conserved β-galactoside-binding lectin that can mediate immune cell homeostasis and acute and chronic inflammation by blunting proinflammatory cytokine synthesis, engaging T-cell apoptotic programs, promoting the expansion of T regulatory (Treg) cells, and deactivating antigen-presenting cells (33). However, the mechanism of galectin-1 in the progression of CAD remains unclear. Previous studies found that knockout of Lgals1 in mice with absent galectin-1 exhibited enhanced cardiac inflammation, attenuated heart function, and dilated heart chambers after AMI. Elevated serum galectin-1 in CAD patients might reflect the compensation of chronic vascular inflammation. These findings indicated that galectin-1 has potential protective effects against AMI (34, 35). In our study, LGALS1 was significantly downregulated in the plasma of CTO patients and increased dramatically after CTO vessel vascularization. This was possibly related to the high compensatory expression of galectin-1 in chronic inflammation. Our proteomic study also identified another galectin that positively correlated with galectin-1. However, there was a lack of a specific peptide, and we could not distinguish which specific member of the galectin family belonged. IGF2 is crucial in regulating cell proliferation, growth, migration, differentiation, and survival by interacting with several receptors and binding proteins (36). Zaina et al. showed that IGF2 contributed to atherosclerotic lesions by promoting cell differentiation *via* autocrine and paracrine signaling (37). They found that circulating levels of IGF2 did not affect the formation of atherosclerotic lesions, but an increased local expression of IGF2 in smooth muscle cells was associated with local intimal thickening. In this study, it was found that the plasma level of IGF2 in CTO patients was increased compared to that in CON patients and decreased after the vascularization of CTO vessels. However, the mechanism of the changes in circulating IGF2 related to CTO vessel formation and post-PCI is inconclusive; the blood flushing effect might take away IGF2 locally secreted by smooth muscle cells. Additionally, CSF1, LGALS1, galectin, and LPL had a significant positive correlation with each other, while galectin and IGF2 showed a negative correlation. These results suggested that there might be a mutual synergistic effect among CSF1, LGALS1, galectin, and LPL in the progression of CAD. In contrast, galectin and IGF2 might exercise the exact opposite function, which needs further verification. To the best of our knowledge, no study has explored the particular effect of ADH4 and IgH on the pathophysiological progression of CAD. However, it does not prevent plasma ADH4 and IgH levels from being used as potential monitoring markers to evaluate the severity of coronary lesions and the successful reperfusion of CTO vessels. Collectively, these dynamically altered proteins are expected to be potential biomarkers for evaluating the success of CTO vessel revascularization and detecting the recovery of vessel function.

In summary, the quantitative proteomic analysis between patients with coronary CTO and control individuals identified differential protein expression in plasma. We also presented the first plasma proteomics analysis of the CTO vessels at early successful revascularization, different from the molecular alterations manifested in the immediate reperfusion of acute coronary occlusions, which highlighted a dynamic pattern of molecular responses related to biological processes in the extracellular structural organization rather than oxidative stress or chronic inflammatory responses. Seven proteins (ADH4, CSF1, galectin, LPL, IGF2, IgH, and LGALS1) might be dynamically altered in plasma during the pathophysiological progression of CTO vessels and following successful revascularization, which would have the potential for the development of new therapeutic approaches to prevent coronary CTO.

## Data availability statement

The datasets presented in this study can be found in online repositories. The names of the repository/repositories and accession number(s) can be found at: http://www.proteomexchange.org/, PXD034267.

## Ethics statement

The studies involving human participants were reviewed and approved by Institutional Medical Ethics Committee of Renmin Hospital, Wuhan University. The patients/participants provided their written informed consent to participate in this study.

## Author contributions

JL, X-JJ, and XY designed the experiments and analyzed data. X-LW, ZQ, TS, W-GW, and X-XZ helped in the patients' recruitment for this study. JL and Q-HW performed software analyses. JL drafted the first version of this manuscript. X-JJ and XY revised the manuscript. All authors have read and approved the final manuscript.

## Funding

This work was supported by grants from the Special Project of Central Government Guides Local Science and Technology Development of Hubei Province (2019ZYYD062) and the Fundamental Research Funds for the Central Universities (2042021kf0132).

## Conflict of interest

The authors declare that the research was conducted in the absence of any commercial or financial relationships that could be construed as a potential conflict of interest. The reviewer FZ declared a shared parent affiliation with the authors to the handling editor at the time of review.

## Publisher's note

All claims expressed in this article are solely those of the authors and do not necessarily represent those of their affiliated organizations, or those of the publisher, the editors and the reviewers. Any product that may be evaluated in this article, or claim that may be made by its manufacturer, is not guaranteed or endorsed by the publisher.

## References

[1] Stone GW Kandzari DE Mehran R Colombo A Schwartz RS Bailey S. Percutaneous recanalization of chronically occluded coronary arteries: a consensus document: part I. Circulation. (2005) 112:2364–72. 10.1161/CIRCULATIONAHA.104.481283 16216980

[2] Godino C Carlino M Al-Lamee R Colombo A. Coronary chronic total occlusion. Minerva Cardioangiol. (2010) 58:41–60. 20145595

[3] Galassi AR Tomasello SD Crea F Costanzo L Campisano MB Marzá F. Transient impairment of vasomotion function after successful chronic total occlusion recanalization. J Am Coll Cardiol. (2012) 59:711–18. 10.1016/j.jacc.2011.10.894 22340262

[4] Canty JM Suzuki G Banas MD Verheyen F Borgers M Fallavollita JA. Hibernating myocardium: chronically adapted to ischemia but vulnerable to sudden death. Circ Res. (2004) 94:1142–9. 10.1161/01.RES.0000125628.57672.CF 15016734

[5] Bai H Sun K Wu JH Zhong ZH Xu SL Zhang HR. Proteomic and metabolomic characterization of cardiac tissue in acute myocardial ischemia injury rats. PLoS ONE. (2020) 15:e0231797. 10.1371/journal.pone.0231797 32365112 PMC7197859

[6] Tatarkova Z Kovalska M Sivonova MK Racay P Lehotsky J Kaplan P. Tyrosine nitration of mitochondrial proteins during myocardial ischemia and reperfusion. J Physiol Biochem. (2019) 75:217–27. 10.1007/s13105-019-00683-7 31115776

[7] Binek A Fernández-Jiménez R Jorge I Camafeita E López JA Bagwan N. Proteomic footprint of myocardial ischemia/reperfusion injury: Longitudinal study of the at-risk and remote regions in the pig model. Sci Rep. (2017) 7:12343. 10.1038/s41598-017-11985-5 28955040 PMC5617837

[8] Nukala SB Regazzoni L Aldini G Zodda E Tura-Ceide O Mills NL. Differentially expressed proteins in primary endothelial cells derived from patients with acute myocardial infarction. Hypertension. (2019) 74:947–56. 10.1161/HYPERTENSIONAHA.119.13472 31446798

[9] Di Mario C Werner GS Sianos G Galassi AR Büttner J Dudek D. European perspective in the recanalisation of chronic total occlusions (CTO): consensus document from the EuroCTO Club. EuroIntervention. (2007) 3:30–43. 19737682

[10] Morino Y Abe M Morimoto T Kimura T Hayashi Y Muramatsu T. Predicting successful guidewire crossing through chronic total occlusion of native coronary lesions within 30 minutes: the J-CTO (Multicenter CTO Registry in Japan) score as a difficulty grading and time assessment tool. JACC Cardiovasc Interv. (2011) 4:213–21. 10.1016/j.jcin.2010.09.024 21349461

[11] Gensini GG. A more meaningful scoring system for determining the severity of coronary heart disease. Am J Cardiol. (1983) 51:606. 10.1016/S0002-9149(83)80105-2 6823874

[12] Bruderer R Bernhardt OM Gandhi T Miladinović SM Cheng L-Y Messner S. Extending the limits of quantitative proteome profiling with data-independent acquisition and application to acetaminophen-treated three-dimensional liver microtissues. Mol Cell Proteomics. (2015) 14:1400–10. 10.1074/mcp.M114.044305 25724911 PMC4424408

[13] Jiang D-S Zeng H-L Li R Huo B Su Y-S Fang J. Aberrant epicardial adipose tissue extracellular matrix remodeling in patients with severe ischemic cardiomyopathy: insight from comparative quantitative proteomics. Sci Rep. (2017) 7:43787. 10.1038/srep43787 28256566 PMC5335613

[14] Götz S García-Gómez JM Terol J Williams TD Nagaraj SH Nueda MJ. High-throughput functional annotation and data mining with the Blast2GO suite. Nucleic Acids Res. (2008) 36:3420–35. 10.1093/nar/gkn176 18445632 PMC2425479

[15] Guo Z-P Hou H-T Jing R Song Z-G Liu X-C He G-W. Plasma protein profiling in patients undergoing coronary artery bypass grafting surgery and clinical significance. Oncotarget. (2017) 8:60528–38. 10.18632/oncotarget.16366 28947991 PMC5601159

[16] McTaggart F Jones P. Effects of statins on high-density lipoproteins: a potential contribution to cardiovascular benefit. Cardiovasc Drugs Ther. (2008) 22:321–38. 10.1007/s10557-008-6113-z 18553127 PMC2493531

[17] Sawada N Obama T Mizuno M Fukuhara K Iwamoto S Aiuchi T. Transfer and enzyme-mediated metabolism of oxidized phosphatidylcholine and lysophosphatidylcholine between low- and high-density lipoproteins. Antioxidants. (2020) 9:1045. 10.3390/antiox9111045 33114515 PMC7712993

[18] Faria SS Fernandes PC Jr Silva MJ Lima VC Fontes W Freitas R. The neutrophil-to-lymphocyte ratio: a narrative review. Ecancermedicalscience. (2016) 10:702. 10.3332/ecancer.2016.702 28105073 PMC5221645

[19] Kim JH Lim S Park KS Jang HC Choi SH. Total and differential WBC counts are related with coronary artery atherosclerosis and increase the risk for cardiovascular disease in Koreans. PLoS ONE. (2017) 12:e0180332. 10.1371/journal.pone.0180332 28753607 PMC5533311

[20] Tsimikas S Lau HK Han K-R Shortal B Miller ER Segev A. Percutaneous coronary intervention results in acute increases in oxidized phospholipids and lipoprotein(a): short-term and long-term immunologic responses to oxidized low-density lipoprotein. Circulation. (2004) 109:3164–70. 10.1161/01.CIR.0000130844.01174.55 15184281

[21] Fefer P Tsimikas S Segev A Sparkes J Otsuka F Otsuma F. The role of oxidized phospholipids, lipoprotein (a) and biomarkers of oxidized lipoproteins in chronically occluded coronary arteries in sudden cardiac death and following successful percutaneous revascularization. Cardiovasc Revasc Med. (2012) 13:11–9. 10.1016/j.carrev.2011.08.001 22079685

[22] Lugrin J Rosenblatt-Velin N Parapanov R Liaudet L. The role of oxidative stress during inflammatory processes. Biol Chem. (2014) 395:203–30. 10.1515/hsz-2013-0241 24127541

[23] Li X Zhou H Guo D Hu Y Fang X Chen Y. Oxidative stress and inflammation: Early predictive indicators of multiple recurrent coronary in-stent chronic total occlusions in elderly patients after coronary stenting. IUBMB Life. (2020) 72:1023–33. 10.1002/iub.2239 32022379

[24] Wagenseil JE Mecham RP. Vascular extracellular matrix and arterial mechanics. Physiol Rev. (2009) 89:957–89. 10.1152/physrev.00041.2008 19584318 PMC2775470

[25] Sapienza P Mingoli A Borrelli V Brachini G Biacchi D Sterpetti AV. Inflammatory biomarkers, vascular procedures of lower limbs wound healing. Int Wound J. (2019) 16:716–23. 10.1111/iwj.13086 30773823 PMC7948871

[26] Hosseini M Ehrhardt N Weissglas-Volkov D Lai C-M Mao HZ Liao J-L. Transgenic expression and genetic variation of Lmf1 affect LPL activity in mice and humans. Arterioscler Thromb Vasc Biol. (2012) 32:1204–10. 10.1161/ATVBAHA.112.245696 22345169 PMC3331946

[27] Chiu P-L Bierende D Lal N Wang F Wan A Vlodavsky I. Dual effects of hyperglycemia on endothelial cells and cardiomyocytes to enhance coronary LPL activity. Am J Physiol Heart Circ Physiol. (2018) 314:H82–94. 10.1152/ajpheart.00372.2017 28986359

[28] Miyashita Y Shirai K. Clinical determination of the severity of metabolic syndrome: preheparin lipoprotein lipase mass as a new marker of metabolic syndrome. Curr Med Chem Cardiovasc Hematol Agents. (2005) 3:377–81. 10.2174/156801605774322292 16250868

[29] Saiki A Oyama T Endo K Ebisuno M Ohira M Koide N. Preheparin serum lipoprotein lipase mass might be a biomarker of metabolic syndrome. Diabetes Res Clin Pract. (2007) 76:93–101. 10.1016/j.diabres.2006.08.004 16956692

[30] Hamilton JA. GM-CSF in inflammation and autoimmunity. Trends Immunol. (2002) 23:403–8. 10.1016/S1471-4906(02)02260-3 12133803

[31] Sjaarda J Gerstein H Chong M Yusuf S Meyre D Anand SS. Blood CSF1 and CXCL12 as causal mediators of coronary artery disease. J Am Coll Cardiol. (2018) 72:300–10. 10.1016/j.jacc.2018.04.067 30012324

[32] Feldreich T Nowak C Carlsson AC Östgren C-J Nyström FH Sundström J. The association between plasma proteomics and incident cardiovascular disease identifies MMP-12 as a promising cardiovascular risk marker in patients with chronic kidney disease. Atherosclerosis. (2020) 307:11–5. 10.1016/j.atherosclerosis.2020.06.013 32702535

[33] Seropian IM González GE Maller SM Berrocal DH Abbate A Rabinovich GA. Galectin-1 as an emerging mediator of cardiovascular inflammation: mechanisms and therapeutic opportunities. Mediators Inflamm. (2018) 2018:8696543. 10.1155/2018/8696543 30524200 PMC6247465

[34] Chou R-H Huang S-S Kuo C-S Wang S-C Tsai Y-L Lu Y-W. Galectin-1 is associated with the severity of coronary artery disease and adverse cardiovascular events in patients undergoing coronary angiography. Sci Rep. (2020) 10:20683. 10.1038/s41598-020-77804-6 33244142 PMC7692553

[35] Seropian IM Cerliani JP Toldo S Van Tassell BW Ilarregui JM González GE. Galectin-1 controls cardiac inflammation and ventricular remodeling during acute myocardial infarction. Am J Pathol. (2013) 182:29–40. 10.1016/j.ajpath.2012.09.022 23142379 PMC5691326

[36] Bergman D Halje M Nordin M Engström W. Insulin-like growth factor 2 in development and disease: a mini-review. Gerontology. (2013) 59:240–9. 10.1159/000343995 23257688

[37] Zaina S Pettersson L Ahrén B Brånén L Hassan AB Lindholm M. Insulin-like growth factor II plays a central role in atherosclerosis in a mouse model. J Biol Chem. (2002) 277:4505–11. 10.1074/jbc.M108061200 11726660

